# Effect of Agroecological Conditions on Biologically Active Compounds and Metabolome in Carrot

**DOI:** 10.3390/cells10040784

**Published:** 2021-04-01

**Authors:** Martin Koudela, Vera Schulzova, Ales Krmela, Hana Chmelarova, Jana Hajslova, Cenek Novotny

**Affiliations:** 1 Department of Horticulture, Czech University of Life Sciences, Kamycka 129, 165 21 Prague 6, Czech Republic; koudela@af.czu.cz; 2 Department of Food Analysis and Nutrition, University of Chemistry and Technology, Technická 5, 166 28 Prague 6, Czech Republic; vera.schulzova@vscht.cz (V.S.); ales.krmela@vscht.cz (A.K.); hana.chmelarova@vscht.cz (H.C.); jana.hajslova@vscht.cz (J.H.); 3 Institute of Microbiology of the Czech Academy of Sciences, Videnska 1083, 142 20 Prague 4, Czech Republic

**Keywords:** carrot cultivars, metabolomic fingerprinting, 6-methoxymellein, ascorbic acid, carotenes, spontaneous infection, farming conditions

## Abstract

Carrot serves as a source of health-beneficial phytochemicals for human diet whose content is affected by agroecological conditions. The effect of conventional, integrated and organic farming on ascorbic acid (AA) and α,β-carotene levels of new carrot cultivars Cortina F1 and Afalon F1 was investigated and their metabolomic profiles were measured by direct analysis in real time ion source coupled with a high-resolution mass spectrometer (DART-HRMS). Cortina and Afalon exhibited high levels of AA and total carotenes under all agroecological conditions tested that fluctuated in broad ranges of 215–539 and 173–456 mg AA.kg^−1^ dry biomass and 1069–2165 and 1683–2165 mg carotene.kg^−1^ dry biomass, respectively. The ratio of β- to α-carotene in both cultivars was about 1.3. The most important variable for the PCA and the partial least squares discriminant analysis (PLS-DA) models for ethyl acetate extracts measured in positive and negative ionization mode was 6-methoxymellein (6-MM). Total carotene content and 6-MM levels were higher in the organic carrot compared to the conventional one and were correlated with a higher level of spontaneous infection. Other important compounds identified were sitosterol, hexose and various organic acids including antioxidant ferulic and coumaric acids. The findings allow comparison of metabolomic profiles and the AA and carotene contents of both cultivars with those of other commercially used carrots.

## 1. Introduction

The carrot (*Daucus carota* L.) is an important vegetable plant produced worldwide, used in human nutrition, serving as an important source of phytochemicals with beneficial effects on human health, namely ascorbic acid (AA), phenolics, carotenes, and polyacethylenes [1]. The importance of carrot in the Czech diet is reflected by the fact that, in 2019 carrot represented 7.5% of the total vegetable sown area in the Czech Republic [2].

The genetic background is an important factor controlled by the producer that modifies the nutritional, sensory, and health-related properties of carrots [3,4,5]. Different carrot cultivars show a wide range of AA content ranging from 21 to 775 mg kg^−1^, dark orange carrots containing four times more AA than yellow, purple, and orange ones [6,7,8]. The amount of AA in carrots depends also on the CO_2_ concentration, storage temperature, and thermal processing method [1]. Dietary provitamin A carotenes are a major source of human vitamin A. The carrot contains 160 to 380 µg g^−1^ fresh biomass carotenes, β-carotene making up some 80% of total carotenoids [9,10]. Carotene content decreases in the order of purple, orange, yellow, and white carrot varieties [11,12]. A 100-fold difference in the content of β-carotene was found between yellow- and orange types of carrot. Carotene content is affected by genetic and environmental factors including soil conditions [13,14,15]. A drop of the average season temperature by 2 °C between the following crop years decreased the carotene levels [16]. Carrots are also rich in phenolic acids and anthocyanins, whose positive health impacts result from their antioxidant activity [17]. Fresh purple and orange cultivars have been reported to have 102 and 35 mg of polyphenols in 100 g of material, respectively [18]. Fertilizer application and various biotic and abiotic stress factors including various elicitors influence the amount of phenolic compounds [19,20,21].

Conventional production aims at maximal crop yields and economical effectivity using industrial fertilizers and pesticides, whereas organic farming provides lower yields of high-quality vegetables and fruits without using agrochemicals [22,23]. Integrated systems produce quality vegetables using ecologically acceptable methods that minimize the input of agrochemicals [24,25]. The amount of bioactive secondary metabolites in plants is affected by differences between organic and conventional growth, especially by soil fertility management [26]. Inconsistent results have been reported concerning the effect of the farming system on the content of nutraceuticals. Sink et al. (2017) [27] reported that organic farming significantly increased the AA content in carrot roots compared to integrated farming and the effect was cultivar-specific. On the other hand, insignificant differences in α-and β-carotene content were observed between the two systems, but the ratio of β- to α-carotene was slightly higher for the roots collected from the integrated system. Similarly, carotene concentration was not different between conventional and organic growth, but a year-to-year variation substantially affected the carotene content [26,28]. A review by Seljasen et al. (2013) [5] comparing several studies on carrot concluded that the amounts of carotenes, AA, polyacetylenes, and 5-*O*-caffeoylquinic acid are only slightly different when organically grown carrot is compared with the conventionally grown one.

Reports on the connection between a farming system and disease occurrence provide ambiguous information. Transcriptome changes occur in potato tubers in response to organic management showing transcriptional induction of the phenylpropanoid pathway linked to *Phytophtora infestans* infection [29]. In organic systems, potato tuber disease occurrence was found to be significantly higher, for example, common scab and late potato blight [30,31,32]. However, the opposite was reported for potato silver scurf infection, where the numbers of tubers infected in the organic system were lower [32]. The soft rot infection caused by *Pectobacterium* spp. was shown not to be influenced by the farming system [32]. The incidence of *Fusarium* fungi in wheat was similar in the conventional and organic systems, whereas in maize the infection in organic samples significantly surpassed that in conventional ones. The results were strongly influenced by weather conditions [33]. Leaf blight of carrot is mostly caused by the fungi *Cercospora carotae* and *Alternaria dauci* and by the bacterium *Xanthomonas campestris* pv. *carotae* and a spontaneous infection evaluated by lesions on carrot leaves can be attributed to these phytopathogens. Moisture is essential for infection because fungal spores and bacterial cells require surface moisture and warm temperatures (25–30 °C) to germinate [34,35].

The appearance of 6-methoxymellein (6-MM) has been linked with pre- and postharvest infections of carrot by various fungal pathogens, such as *Alternaria* species, *Botrytis cinerea*, *Cercospora carotae* and *Mycocentrospora acerina* [36,37,38,39], with a field attack by carrot psyllid (*Trioza apicalis* Förster) [5], and also with an abiotic stress caused by UV radiation [40,41] or ethylene [42,43]. It was also described as a natural fungal metabolite [44]. The phytoalexin 6-MM inhibits fungal and bacterial growth and conidia germination in various *Alternaria* species and accumulates in carrot roots and leaves in response to fungal infection [38,45,46,47]. The 6-MM accumulation is probably triggered by extracellular pectinolytic enzymes secreted by phytopathogenic fungi [48]. The carrot pathogen *Alternaria radicina* was reported to induce 6-MM accumulation more efficiently than *A. brassicicola* [42], whereas another study did not find any significant increase in 6-MM in several carrot cultivars in response to infection by *A. radicina* or *A. alternata* [49]. Further investigation is needed to specify the 6-MM role in fungal pathogen infections of carrot. 6-MM is, like other phytoalexins, biologically active toward a wide range of organisms and was considered to be an anticancer agent that inhibits breast cancer stem cell [50].

The aim was to investigate the effect of the farming system on the levels of AA and α- and β-carotenes in four newly bred carrot cultivars used for commercial production in central and eastern Europe. The contents of AA and carotenes were compared in organic, conventional and integrated systems. Moreover, the metabolomic fingerprinting was measured by direct analysis in real time ion source coupled with a high-resolution mass spectrometer (DART-HRMS) for examination of the influence of various factors on the carrot metabolome, for example, to see the difference between the three production systems. 6-MM was an important marker whose levels were influenced by the crop year, cultivar, and the farming system. The effect of farming systems and growth density on the level of spontaneous infection were investigated and the results correlated with the presence of 6-MM. Behavior of carrot cultivars in different conditions for two subsequent crop years documenting the importance of the individual factors impacting on the content of health-related compounds represents novel findings that make possible their comparison with other cultivars used for commercial production.

## 2. Materials and Methods

### 2.1. Biological Material

The carrot (*Daucus carota* L.) cultivars were obtained from Moravoseed a.s. (Mušlov, Czech Republic) where they were bred. Afalon F1 is a semi-late hybrid cultivar of the transition type suitable for mechanized harvest whose vegetation time is 115–120 days. Cortina F1 is a late hybrid cultivar of the Flakkee type suitable for mechanized harvest whose vegetation time is 150–160 days. Aneta F1 is a semi-early hybrid cultivar of the Nantes type suitable for early spring or early summer seeding whose vegetation time is 100–105 days. Marion F1 is an early hybrid cultivar of the Nantes type suitable for all-year seeding whose vegetation time is 90–95 days. All varieties are exported and widely used in Eastern Europe.

### 2.2. Production Systems

The experiments were conducted in two localities: Troja (Prague) and Svijanský Újezd. The experimental station of the Czech University of Life Sciences, Prague in Troja has an altitude of 195 m above sea level and the locality is characterized as a dry, medium-warm one with a soil classified as modal fluvisol (pH 6.9). The characteristic of the locality Svijanský Újezd was the following: altitude of 269 m above sea level, sandy loam soil, pH 6.9–7.4. The climatic conditions monitored in the years 2012 and 2013 and compared to the 30-year average temperature and precipitation profiles of the site are shown in Supplementary Materials (Figures S1–S4).

The conventional system (Troja, Svijanský Újezd, Czech Republic) used the following fertilization conditions: 120 kg nitrogen per ha (urea before seeding 80%, ammonium nitrate with limestone (LAV, Agro CS, Říkov, Czech Republic), after sprouting, 20%). The chemical protection was (per ha): Bandur (Bayer CropScience, Frankfurt/Main, Germany) 2 L, Garland Forte (Dow AgroSciences, Prague, Czech Republic) 1.5 L, Ortiva (Syngenta Limited, Cambridge, UK) 1 L. In the integrated system in Troja, the fertilization conditions were similar as in the conventional system. However, the chemical protection was ensured by using (per ha) Stomp (BASF AG, Agricultural Products, Ludwigshafen/Rhein, Germany) 3.5 L and Ortiva (Syngenta Limited) 1 L. In the organic system (Troja), no chemical protection was used and the fertilization conditions included the use of 42 kg nitrogen per ha in the form of Organica (Agro CS) organic fertilizer.

At the Troja experimental plots, the sowing densities were 6 × 10^5^ and 9 × 10^5^ seeds ha^−1^, whereas at Svijanský Újezd only the sowing density of 9 × 10^5^ seeds ha^−1^ was used. Each cultivar in every crop system was grown in four replicates. The size of the experimental plot for each replicate was 9 m^2^. From each replicate, three carrot roots were collected, which made (per variant) 3 roots × 4 replicates = 12 roots. This applied for each cultivar and each crop system.

### 2.3. Spontaneous Infection

The method used for the evaluation of spontaneous infection was based on the protocol of Pawelec et al. (2006) [51] measured the proportion of infected leaves on individual plants. The empirical evaluation scale was the following: 0: no leaves infected; 1: <5% leaves infected; 3: 5–30% leaves infected; 5: 30–60% leaves infected; 7: 60–90% leaves infected; 9: >90% leaves infected or a majority of leaves came off. Each sampling included four independent parallels.

### 2.4. Chemical Analyses

#### 2.4.1. AA Determination

The extraction method was modified for the carrot matrix [52]. An amount of 30 g of fresh carrot biomass was extracted with 60 mL of metaphosphoric acid (Fluka, Buchs, Switzerland) (3%; *w*/*w*) by homogenization in a laboratory blender (Ultra-Turrax IKA-T10 basic, IKA, Staufen, Germany) at the laboratory temperature. The homogenate was filtered, adjusted to a volume of 100 mL and filtered again (5 μm membrane filter, Chromservis, Prague, Czech Republic) into vials. AA was detected using HPLC with UV detection (liquid chromatograph HP 1200 with DAD detector, Agilent Technologies, Santa Clara, CA, USA). The conditions were: LiChroCART, LiChrospher 100 RP-18 (Merck, Darmstadt, Germany) chromatographic column (125 × 4 mm, particle size 5 μm) with precolumn (4 × 4 mm, particle size 5 μm); mobile phase 5% methanol (gradient grade, Sigma-Aldrich, Taufkirchen, Germany) (*v*/*v*), pH = 3 (ortho-Phosphoric acid, Lachner, Neratovice, Czech Republic; deionized water (18 MΩ cm) was produced by a Milli-Q system (Millipore, Darmstadt, Germany); flow rate 0.8 mL min^−1^; temperature 35 °C; injection volume 5 μL; UV detection at 244 nm.

AA was identified by comparing the retention times of the individual samples with those of a standard (l-ascorbic acid, Sigma-Aldrich, Taufkirchen, Germany, purity ≥ 99%). For quantification, an external calibration curve was used. The method was validated and the repeatability expressed as relative standard deviation (RSD = 5%, calculated from 6 parallel measurements), the LOQ was found to be 5 mg kg^−1^ and the recovery 95%.

Dry carrot biomass was prepared by drying at 105 °C for 5 h and used to express the AA and carotene contents per kg dry mass. The results were statistically processed by *t*-test and analysis of variance (ANOVA) at a significance level *α* = 0.05.

#### 2.4.2. Carotene Determination

The α- and β-carotene content was analyzed in fresh carrot samples using a modified method of Bhave et al. (2017) [53]. The carotenes were extracted by an ethanol/hexane mixture (1:1; *v*/*v*) containing 0.2 % tert-butyl-hydroxytoluene (BHT) and annealed sodium sulfate (Lachner, Neratovice, Czech Republic) (3 g per 1 g biomass) under shaking (30 min) followed by centrifugation (10,000 rpm, 5 min). Ethanol and *n*-hexane were purchased from Merck KGaA (Darmstadt, Germany), BHT from Sigma-Aldrich (Taufkirchen, Germany). The upper hexane layer was removed and the ethanolic portion was reextracted with hexane three times. The combined hexane extracts were evaporated at 30 °C and the dry residue dissolved in an acetone/ethanol (4:6; *v*/*v*) mixture containing 0.2% BHT and subsequently filtered (0.22 μm centrifugation PTFE microfilters, Chromservis, Praha, Czech Republic) into a vial. The samples were protected against light during the extraction, stored in dark vials, and analyzed immediately after the extraction. An Agilent Technologies 1200 HPLC with DAD detector (liquid chromatograph HP 1200, Agilent Technologies, Santa Clara, CA, USA) device equipped with a visible-light photodiode array detector was used to analyze the samples. The chromatographic separation was carried out using an analytical column Poroshell 120 EC-C18; 100 × 2.1 mm; particle size 2.7 µm (Agilent Technologies, Santa Clara, CA, USA) at 30 °C column temperature. The mobile phases were (A) water and (B) methanol (MeOH), with gradient elution and a flow rate of 0.5 mL min^−1^. The DAD measurements were recorded in the UV and visible regions (190 to 800 nm), with the quantification at 450 nm for α- and β-carotene.

The β-carotene standard (purity ≥ 99 %, Sigma-Aldrich, Taufkirchen, Germany) was prepared by dissolving an amount of 4–5 mg β-carotene in 25 mL of the acetone/ethanol (4:6, *v*/*v*) mixture containing 0.2% BHT and the solution was used for preparation of a calibration curve in the range of 0.2–20 μg mL^−1^. The LOQ was 5 mg kg^−1^. The method was validated and the repeatability expressed as the relative standard deviation (RSD = 3% for α- and β-carotene, calculated from 6 parallel measurements). Because of the instability of β-carotene, a fresh standard was prepared before each measurement and protected against light. The α-carotene content was quantified using the β-carotene standard. The results were statistically processed by *t*-test and analysis of variance (ANOVA) at a significance level *α* = 0.05.

### 2.5. Metabolomic Fingerprinting

An amount of 5 g of the homogenized sample was extracted with 5 mL of a solvent (methanol for polar compounds and ethylacetate for low polar compounds extraction; both were from Sigma-Aldrich, Prague, Czech Republic). under vigorous shaking for 2 min and subsequently centrifuged (10,000 min^−1^, 10 min; Rotina 38R, Hettich Zentrifugen, Tuttlingen, Germany). The upper layer was then collected for the DART–MS analysis. Every sample was analyzed in four replicates (each replicate was made from at least 3 carrot roots).

#### 2.5.1. DART-OrbitrapMS Method

The DART–HRMS system consisted of the DART-SVP ion source with an XZ transmission module autosampler (IonSense, Saugus, MA, USA) coupled to the Exactive benchtop mass spectrometer (Thermo Fisher Scientific, Waltham, MA, USA). A Vapur interface (IonSense, Saugus, MA, USA) was employed to hyphenate the ion source and the mass spectrometer. The distance between the exit of the DART gun and the ceramic transfer tube of the Vapur was set to 10 mm, the gap between the ceramic tube and the inlet to the heated capillary of the Exactive was 2 mm. The DART–HRMS instrument was operated either in positive or negative ionization mode, the optimized settings of the system parameters were as follows: (i) DART ionization: helium pressure 4 bar; gas temperature 350 °C (MeOH extracts)/450 °C (EtAc extracts); discharge needle voltage: 5000 V; grid electrode: ±350 V; (ii) mass spectrometric detection: capillary voltage: ±60 V; tube lens voltage: ±120 V; capillary temperature: 250 °C. Sheath, auxiliary and sweep gases were disabled during DART–HRMS analysis. The mass spectrometer was operated at mass resolving power 50,000 full width at half maximum (FWHM) calculated for *m*/*z* 200. Under these settings, the mass spectra acquisition rate was 2 spectra s^−1^. The monitored mass range was 55–1100 *m*/*z*. Sample extracts (3 µL) were put on the XZ transmission module grid. The desorption and gap times were 7 and 5 s, respectively.

#### 2.5.2. Chemometric Analysis

Potential markers were selected after careful examination of the measured DART-MS spectra; the ions with significant intensities (threshold 2% of the relative ion intensity) were chosen and ions with *m*/*z* belonging to ^13^C isotopes were excluded. The raw data in the form of absolute peak intensities (heights) were normalized using the constant row sum, that is, each variable was divided by the sum of all variables for each sample. This procedure transformed all data to a uniform range of variability.

The SIMCA software (v. 13.0, 2011, Umetrics, Sweden) was used for multivariate data analysis. Pareto scaling (square root of the standard deviation was used as the scaling factor) was applied prior to the analysis. The principal components analysis (PCA) was employed as an unsupervised chemometric technique for the first view into a data structure; partial least squares discriminant analysis (PLS-DA) was used as a supervised technique for the classification of samples according their classes (crop year, variety, way of farming). Variables important for separation were selected and, although the primary task was untargeted fingerprinting, some of the variables were tentatively identified.

## 3. Results and Discussion

### 3.1. Effect of Farming System on AA Content of Carrot Cultivars

The respective values of dry biomass of Cortina and Afalon cultivars grown in the conventional, integrated and organic farming systems fluctuated between 11.9% and 14.7% and 11.0% and 14.1%, irrespective of the year and the seeding density used. This is in agreement with other studies that reported values between 11% and 14% [54].

**Figure 1:**
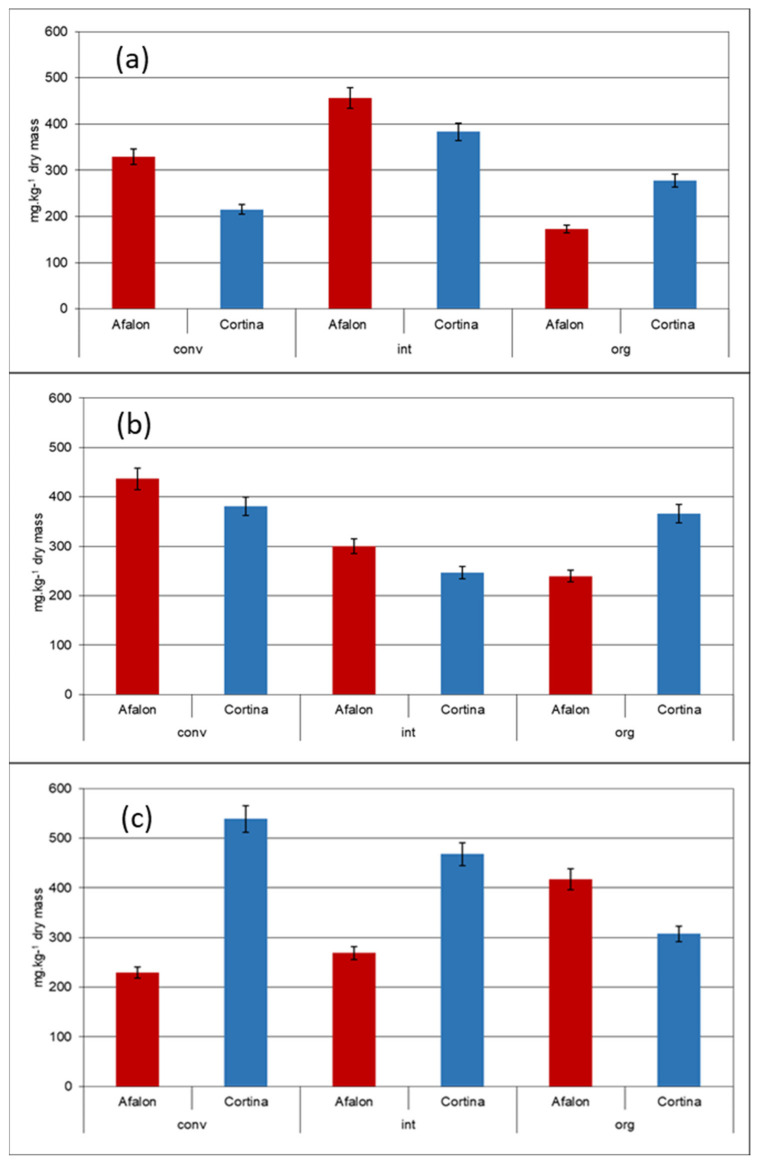
Comparison of AA content in Cortina (blue) and Afalon (red) grown in conventional (conv), integrated (int) and organic (org) systems: (**a**) crop year 2012, seeding density 9 × 10^5^ seeds ha^−1^; (**b**) crop year 2013, seeding density 6 × 10^5^ seeds ha^−1^; and (**c**) crop year 2013, seeding density 9 × 10^5^ seeds ha^−1^.

The AA levels of 215–539 and 173–456 mg kg^−1^ dry weight measured in Cortina and Afalon cultivars in 2012 and 2013 in different production systems (Figure 1a,b,c) were higher than those of semi-late and late carrot cultivars reported by Bratu et al. (2006) [55] and Matějková and Petříková (2010) [16] but comparable to the contents reported by Faisal et al. (2017) [56] for winter-season grown carrots. The content of AA was affected by the farming systems used. In 2012, the highest levels of AA in both cultivars were found in the integrated-system-grown carrots (Afalon, 456 ± 23 mg kg^−1^; Cortina, 383 ± 19 mg kg^−1^). Relatively low respective levels of 173 ± 9 and 278 ± 14 mg kg^−1^ were detected in the organically produced carrots. Those observations were not confirmed in the 2013 crop where the maximal AA contents were detected in conventional growth samples in both cultivars (Figure 1b,c). Our results document the importance of the year effect (cf. also PCA score scatter plot in Figure 4) demonstrated for quality characteristics of carrot by Seljasen et al. (2012) [57]. The changes in the AA content were not correlatable with a higher biological stress in the organic system caused by a higher spontaneous infection (Figure 6) as reported for some phytonutrients by Cwalina-Ambroziak et al. (2014) [4] and Perrin et al. (2017) [58]. The observed effect of the organic system was thus different from that recorded with cabbage where the content of AA in the organically produced plants was up to 2.2-fold compared to the integrated system-grown plants [59].

In 2012, the Afalon cultivar exhibited higher AA levels compared to Cortina in the conventional and integrated systems, whereas it was opposite in the organic system (Figure 1a). Similar results were obtained also for the 2013 crop year when the seeding density of 6 × 10^5^ plants ha^−1^ was used (Figure 1b). This observation was not confirmed using the seeding density of 9 × 10^5^ plants ha^−1^ (Figure 1c). The two cultivars thus behaved differently when comparing their AA contents when the seeding densities 6 × 10^5^ and 9 × 10^5^ seeds ha^−1^ were used. These observations confirm a significant variability of AA contents in various cultivars reported also by other authors [1,11,16]. No predominant effect of the farming system or seeding density on AA content was observed in Afalon or Cortina cultivars. Similarly, Fjelkner-Modig et al. (2001) [60] concluded that no significant differences due to the growth system were noticed for AA in various vegetables including carrot. Large fluctuation of the AA content was observed in both cultivars in 2012 and 2013 (Figure 1a–c) and overall, there were no statistically significant trends in the AA content (*t*-test; *α* = 0.05).

### 3.2. Effect of Farming System on the Content of Carotenes in Carrot Cultivars

**Figure 2:**
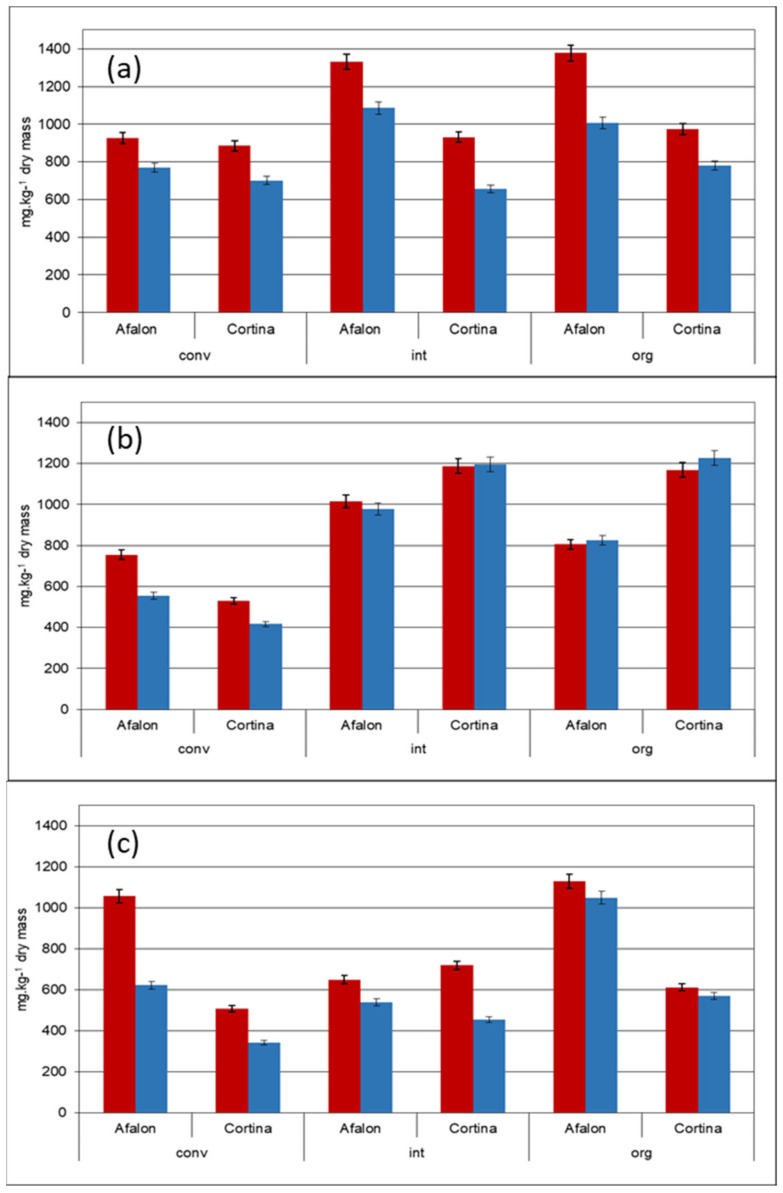
Comparison of β-carotene and α-carotene content in Cortina and Afalon grown in conventional (conv), integrated (int) and organic (org) systems: (**a**) crop year 2012, seeding density 9 × 10^5^ seeds ha^−1^; (**b**) crop year 2013, seeding density 6 × 10^5^ seeds ha^−1^; (**c**) crop year 2013, seeding density 9 × 10^5^ seeds ha^−1^. The content of carotenes is expressed in mg per kg dry weight; β-carotene, red; α-carotene, blue.

The total content of carotenes was higher in Afalon compared to Cortina (*p* = 0.0467), the respective average contents in the three farming systems in 2012 were 2165 ± 108 and 1641 ± 82 mg kg^−1^ dry biomass (Figure 2a). The seeding density used in 2012 was 9 × 10^5^ seeds per ha. In 2013, this difference between the cultivars was confirmed for carrots grown at the seeding density of 9.10^5^ seeds per ha (*p* = 0.0467) (Afalon 1683 ± 84 mg kg^−1^ dry biomass, Cortina 1069 ± 53 mg kg^−1^ dry biomass) but not for those growing at 6 × 10^5^ seeds per ha (Afalon 1644 ± 82 mg kg^−1^ dry biomass, Cortina 1908 ± 95 mg kg^−1^ dry biomass) (Figure 2b,c). An effect of seeding density on the carotene content was reported, probably due to the action of carotenoids as photoprotection compounds where high intensity of UV light may cause an increase in carotene content [5,61,62]. This is in agreement with our results, especially in Cortina cv., when we observed that the carotenes level (in particular α-carotene) was higher at the lower seeding density in all three production systems. On the other side, the response was less clear-cut in Afalon cv. (Figure 2b,c). Probably, various cultivars may respond differently.

The above carotene levels were superior to those found in Finnish carrot cultivars (81 mg kg^−1^ fresh biomass [63], in five different early-to-late Czech cultivars (84–141 mg kg^−1^ fresh biomass, [16], or in 8 different Turkish cultivars [3]. Significant effect of the year when carrot was produced on the content of carotenes was reported by Simon and Wolff (1987) [64] and Soltoft et al. (2010) [20].

When assessing the effect of the farming system, the total content of carotenes was found significantly higher in organically and integrated grown carrot compared to the conventionally grown one (*p* = 0.00783), irrespective of the year, cultivar, or seeding density used (Figure 2a–c). A higher level of spontaneous infection was detected on carrot leaves in the organic and integrated farming system compared to the conventional one (Figure 6). However, whether the increased infection could affect the carotene level remains unclear, even though there are indications that biological stress can influence the carotene content in carrot roots [55]. In contrast, comparative studies suggest a rather nonsignificant effect of the cultivation system on the carotene levels [5,20,65,66].

Under most farming conditions, the content of β-carotene exceeded that of α-carotene, the ratio of 1.3 was detected in both cultivars (Figure 2a–c). A higher content of β-carotene to α-carotene is in agreement with other works concerning carrot [28,67,68]. Figure 2c shows that, in 2013, a higher ratio of β- to α-carotene was often observed (Figure 2b,c), compared to 2012 (Figure 2a). Our results confirm the observation of Paoletti et al. (2012) [69] who documented that the content of β-carotene can differ by 24% between individual crop years. Similarly, other studies brought evidence on the effect of the crop year on the carotene content of potato tubers and Hokkaido-type pumpkin attributing the effect to the growth temperature [70,71]. The average temperatures during the vegetation period of 2013 were up to 5 °C higher compared to the 30-year average value (Figures S1 and S2), so we can speculate that the growth temperature significantly influenced the β- to α-carotene ratio, perhaps by affecting the activities of phytoene synthase ε-lycopene cyclase in carotene biosynthesis [72,73,74,75]. When grown organically, the β- to α-carotene ratio was close to 1 in both cultivars (Figure 2a–c). Generally, the results confirm a high variability of the carotenes content documented by other authors [3,11]. The statistical results obtained by *t*-test are summarized in Table 1.

### 3.3. Effect of Locality on AA and Carotenes Content of Carrot Cultivars

**Figure 3:**
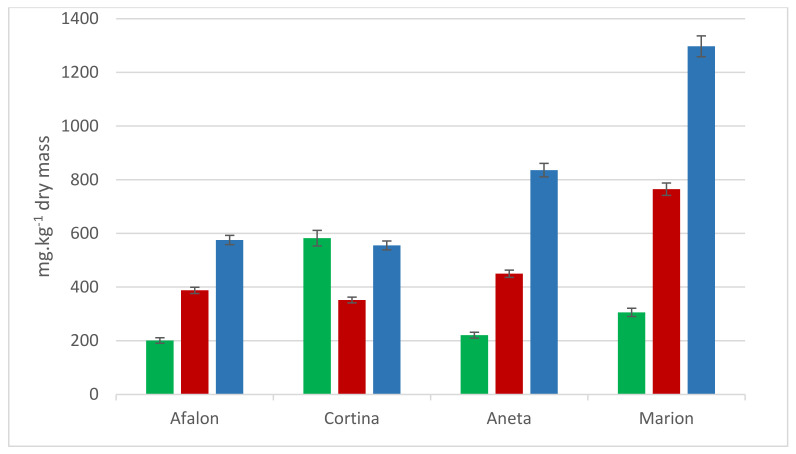
Comparison of AA, β-carotene and α-carotene content in Marion, Aneta, Cortina and Afalon cv. grown in conventional system in crop year 2013, seeding density 9 × 10^5^ seeds per ha in mg per kg dry weight; AA, green; β-carotene, red; α-carotene, blue.

Four varieties were conventionally grown in the locality Svijanský Újezd at the seeding density of 9 × 10^5^ seeds per ha in 2013. Content of AA and carotenes (Figure 3) was compared with conventionally grown carrots from the locality of Troja (see Figure 2c). AA content was comparable for varieties Afalon and Cortina in both localities; the amount of AA in Cortina was almost three-fold compared to the other cultivars. When the four varieties grown in the locality Svijanský Újezd were compared, the contents of total carotenoids were decreasing in the order Marion > Aneta > Afalon, Cortina (Figure 3).

### 3.4. Metabolomic Profiling

**Figure 4:**
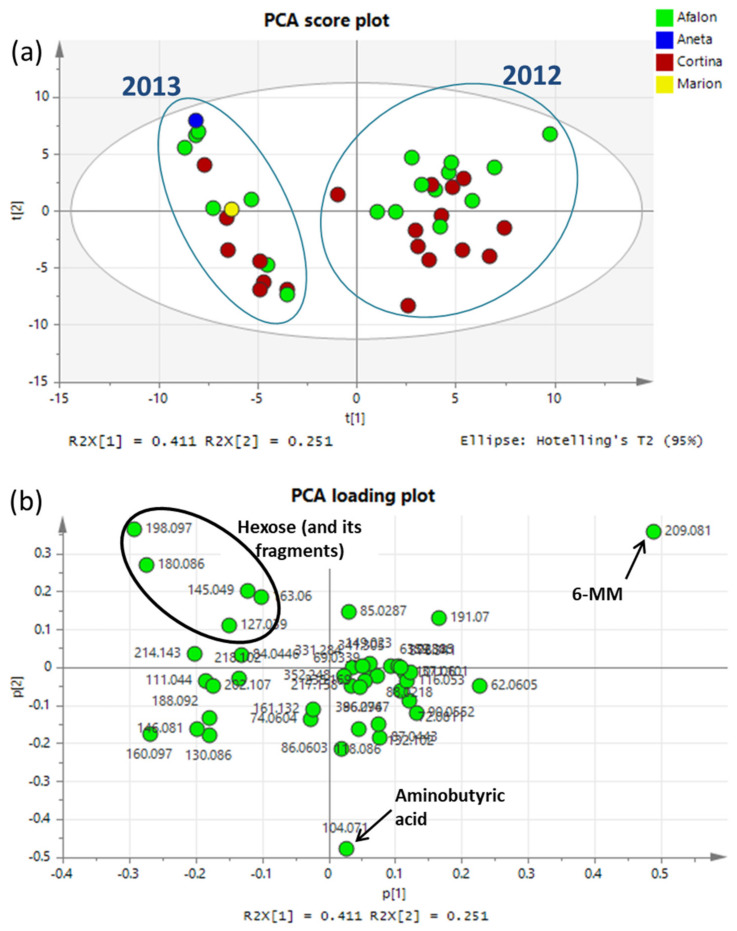
(**a**) The first two principal components analysis (PCA) score scatter plots of the whole set of samples (colored according to the varieties), t[1] is the first principal component (PC), representing 41.1% of the total variance, t[2] is the second PC, representing 25.1% of the total variance; (**b**) PCA loading plot, where positions of features (represented by measured exact mass and respective intensity) explain positions of samples in PCA score plot direct analysis in real time ion source coupled with a high-resolution mass spectrometer (DART(+)-MS) data).

A DART ionization source coupled to a high resolution orbitrap mass spectrometer (DART-OrbitrapMS) was applied for the detection of all ionizable small molecules (metabolomic fingerprinting/profiling). DART is a unique ion source belonging to ambient mass spectrometry ionization techniques, forming ions in open space outside the vacuum system of the mass spectrometer, without previous chromatographic separation [76,77]. The data matrix comprising the DART-MS spectral information (in this case 43 ions characterized by *m*/*z* value and relative intensity) was first processed by principal components analysis (PCA), which is used as an unsupervised technique for the first view into the data structure. The PCA score plot was created based on the first two principal components (PC1 and PC2), which cover 66% of the sample set variability (41% and 25% for PC1 and PC2, respectively). The first five PCs explain 89% of the total variance of the PCA model. The PCA score plot is shown in Figure 4a, from which it is apparent that the major differences in the metabolome are between the crop years 2012 and 2013. Furthermore, a partial trend is evident in the case of varieties, where Afalon samples are located more in the upper part of the plot, while Cortina is more at the bottom. The position of the samples can be explained by the positions of markers in the loading plot (Figure 4b). For example: an important marker, whose levels in the carrot samples were significantly influenced by the crop year, variety and farming system, was identified as 6-methoxymellein (6-MM). This marker is located at the top right in the loading plot, which means that this marker is present at higher levels in the samples located at the top right in the score plot compared to the other samples.

**Figure 5:**
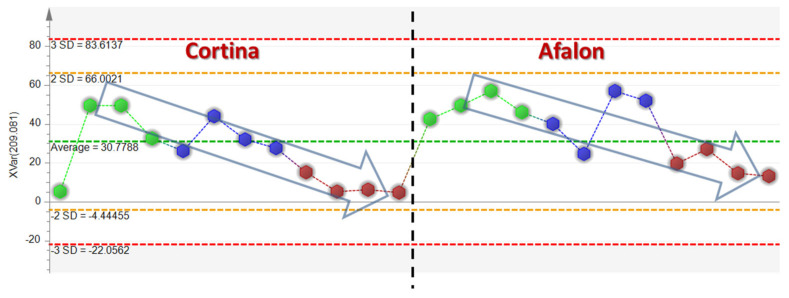
Differences in signal intensities of 6-MM (*m*/*z* = 209.081) in ethylacetate extracts in positive ion mode (organic system–green, integrated system–blue, and conventional system–red).

In the next step of data analysis, PLS-DA models were constructed for further improvement of the separation between the groups of samples. In addition to the differences in metabolome between the crop years, differentiation between the varieties was also observed, and, especially in the year 2012 when significant differences were found between the samples grown conventionally vs. the organic and integrated ones (Figure 5). These revealed differences in metabolomes are discussed in detail in the following section dealing with the identification of markers important for the differentiation of the sample classes. The examples of DART-MS spectra are shown in Supplementary Information (Figures S5 and S6).

#### Marker Identification

The use of the SIMCA software function “Variable importance of the projection” for PLS-DA allowed us to obtain the most important markers for class recognition. Some of these markers were tentatively identified. The molecular formulas were estimated based on (i) accurate mass of the ions (mass error < 3 ppm) and (ii) isotopic profile of the ions, followed by their identification using database search and/or comparison with recently published studies.

**Figure 6:**
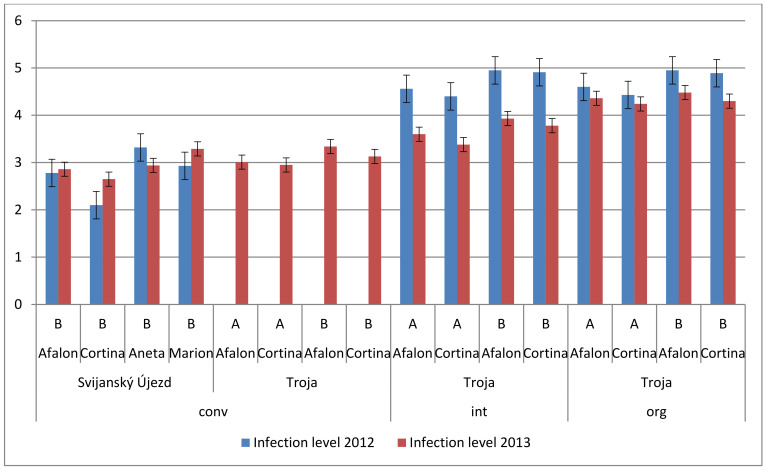
Effect of seeding density and production system on the levels of spontaneous infection of Cortina and Afalon at the Troja locality in 2012 and 2013. Seeding density: A, 6 × 10^5^ seeds ha^−1^; B, 9 × 10^5^ seeds ha^−1^; the level of spontaneous infection is expressed in arbitrary points.

The most important variable for groups separation, specifically for the differentiation between the two crop years and, within the year 2012, for the separation of organic and integrated samples from the conventional ones, was identified as 6-methoxymellein. This difference can relate to a significantly higher spontaneous infection of the organic and integrated production, which were not treated with a fungicide (Figure 6). This infection resulted in a significant induction of 6-MM, which is the most important marker for the differentiation of the samples in relation to the development of the infection. This marker was detected in both positive (*m*/*z* = 209.081) and negative (*m*/*z* = 207.066) ionization modes with the same significance. The identification of 6-MM was supported by liquid chromatography-high resolution tandem mass spectrometry [LC-HRMS/MS, employing an Acquity Ultra-Performance LC system coupled to a Synapt G2 HD spectrometer (Waters, Milford, MA, USA)]. The parent and product mass spectra are shown in Supplementary Information (Figure S7) and the structure was confirmed with a predicted spectrum (obtained by https://hmdb.ca/spectra/ms_ms/9648, accessed on 27 February 2021).

The variation plot in Figure 5 demonstrates the differences in 6-MM levels in the compared groups of analyzed samples for the year 2013. There were no clear differences in 2012 and the relative signals of 6-MM were more than an order of magnitude lower compared to 2013. Studies of other authors confirm this association, namely an induction of 6-MM accumulation in the presence of *Alternaria radicina* and *A. brassicicola* as well as an accumulation of stress-related compounds including 6-MM in carrot roots infected with *Botrytis cinerea* or *Mycocentrospora acerina* [36,39,42].

**Figure 7:**
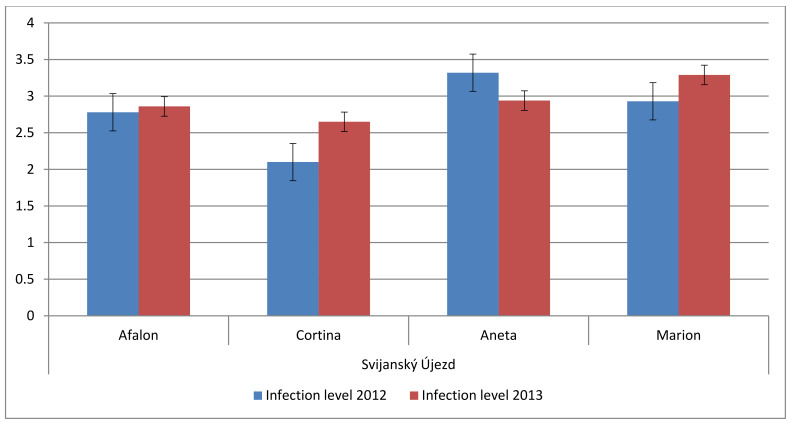
Comparison of spontaneous infection level in various carrot cultivars at the Svijanský Újezd locality, grown conventionally at the seeding density of 9 × 10^5^ seeds ha^−1^ in 2012 and 2013; the level of spontaneous infection is expressed in arbitrary points.

When comparing the relative response intensities for 6-MM in terms of varieties, it was found that Afalon cv. contained almost twice the levels detected in the Cortina cv. In the latter cultivar, there was an approximately 7-fold increase in the response for 6-MM for the integrated cultivation compared to the conventional one and a 9-fold increase for the organic cultivation. As to the Afalon cultivar, the respective increases were only 3- and 3.5-fold. The presence of 6-MM might be associated with the spontaneous infection observed (see Figure 6 and Figure 7).

Other important compounds identified in the extract were sitosterol, carotenes, hexose, and organic acids such as fumaric/malic, linoleic, succinic, ferulic, and coumaric (listed in Supplementary Information Table S1). The latter two belong to a broader group of antioxidant phenolics whose function in the defence against oxidative stress and in the prevention of degenerative diseases was proven [1]. Our results were in accordance with the results of Cwalina-Ambroziak et al. (2014) [4] who measured high concentrations of phenolic compounds in organically grown Koral cultivar carrot roots. In contrast, the conventional system seemed to enhance the overall phenolic–antioxidant richness of sweet peppers but this trend was not observed for all individual phenolics [78].

### 3.5. Effect of Farming System on Spontaneous Infection of Carrot

Spontaneous infection of carrot plants was evaluated by the intensity of leaf surface damage of the carrot plants and compared at two localities and two subsequent years using different farming systems. Carrot leaf blights are mostly caused by the fungal pathogens *Alternaria dauci* and *Cercospora carotae* and by the bacterium *Xanthomonas campestris* pv. *carotae* [35] but we did not distinguish between the effects of the individual pathogens. An empirical evaluation scale was used to quantify the extent of infection by assessing the proportion of infected leaves on individual plants [51]. The data measured in 2013 showed an increasing level of infection in the order conventional < integrated < organic farming in both cultivars, irrespective of the seeding density; that can be explained by the chemical protection used under conventional conditions compared to the organic conditions where no chemical protection was used (Figure 6). This observation confirmed the results obtained previously with carrot and cabbage plants [39,59]. The results obtained in 2012 comparing the infection in the integrated and organic system showed no such difference between the infection levels, suggesting that particular crop year conditions can influence the infection level (Figure 6).

The increase of the seeding density from 6 × 10^5^ to 9 × 10^5^ plants ha^−1^ resulted in enhanced levels of spontaneous infection in both crop years and was observed in both cultivars in all farming systems tested (Figure 6). A higher seeding density evidently resulted in an increased humidity in the plant cover and a rise of leaf moisturizing period, leading to an increase of the infection level. The importance of wet conditions has been reported for the development of bacterial and fungal infections [34,35,79].

Cortina cultivar exhibited a lower susceptibility to spontaneous infection than Afalon, which manifested at both seeding densities (Figure 6). The differences were not statistically significant. Their resistance to spontaneous infection was higher compared to that of the other two carrot cultivars, Aneta and Marion (Figure 7). Differences in resistance to the *Alternaria dauci* infection between individual carrot cultivars were reported by Carvalho et al. (2005) [80]. The sensitivity to infection in resistant cultivars can be lower by 25% compared to that of the sensitive ones [81].

## 4. Conclusions

The effect of different farming systems, crop year and locality on the contents of AA and carotenes, and the level of spontaneous infection in the carrot cultivars Afalon and Cortina were investigated. Crop year factor was found to be dominant with respect to the contents of carotenes and metabolomic fingerprints measured by the DART-MS method. The farming system significantly affected the carotenes content that was found to be higher in organically and integrated grown carrots compared to the conventional ones. The locality did not have much effect on the AA level and the effect was cultivar dependent. PLS-DA models showed that 6-MM was the most important marker differentiating between the crop years as well as between the organically and integrated-grown carrot samples on the one side and the conventionally grown ones on the other side. The 6-MM levels were related to a high spontaneous infection. When comparing carrots from conventional and organic farming, the relative 6-MM response intensities were cultivar specific. Other important compounds identified in the metabolome were sitosterol, hexose and organic acids including ferulic and coumaric acid. Spontaneous infection was lower in the conventional farming system compared to the organic one. Seeding density was an important factor affecting the spontaneous infection on the background of the crop year, cultivar and farming system used. The high phytonutrient quality of Afalon and Cortina carrot cultivars, compared with other commercially used carrots, was demonstrated under various growth conditions.

## Figures and Tables

**Table 1:** An overview of trends in carotene levels in carrots (obtained by *t*-test).

Factor	Trends in Carotenes Content	Degrees of Freedom	*p*-Value
Year	Significantly higher in 2012; a higher ratio of β- to α-carotene in 2013	20	0.0327 *
Cultivar (Cortina vs. Afalon) in 2012	Significantly higher in Afalon	4	0.0467 *
Cultivar (Cortina vs. Afalon) in 2013	Higher in Afalon in some cases, but statistically inconclusive	16	0.1640
Farming system (org. + int. vs. conv)	significantly lower in conventionally grown carrot; significantly higher β- to α-carotene ratio	20	0.00783 *
Seeding density	Higher in 6 × 10^5^ seeds per ha, in particular α-carotene	14	0.0864
Locality (Troja vs. S. Újezd)	No significant differences	6	0.3761

* statistically significant difference at *α* = 0.05.

## Data Availability

Not applicable.

## Supplementary Materials

The following are available online at https://www.mdpi.com/article/10.3390/cells10040784/s1, Figure S1: Average temperature profile (°C) at the trial station Troja during the vegetation period 2012 (red) and 2013 (blue) compared to the 30-year average (green), Figure S2: Average temperature profile (°C) at the trial station Svijanský Újezd during the vegetation period 2012 (red) and 2013 (blue) compared to the 30-year average (green), Figure S3: Average precipitation profile (mm) at the trial station Troja during the vegetation period 2012 (red) and 2013 (blue) compared to the 30-year average (green), Figure S4: Average precipitation profile (mm) at the trial station Svijanský Újezd during the vegetation period 2012 (red) and 2013 (blue) compared to the 30-year average (green), Figure S5: DART-Orbitrap-MS spectra of ethylacetate extracts of Cortina carrot cv. measured in positive ionization mode. Carrot grown in: organic farming system (top), conventional farming system (bottom), Figure S6: DART-Orbitrap-MS spectra of ethylacetate extracts of Cortina carrot cv. measured in negative ionization mode. Carrot grown in: organic farming system (top), conventional farming system (bottom), Figure S7: LC-MS and LC-MS/MS spectrum of 6-methoxymellein (methanolic extract, positive ionization mode), Table S1: Summary of tentatively identified compounds found in carrot extracts.
