# Supplementary material for: Effect of Agroecological Conditions on Biologically Active Compounds and Metabolome in Carrot
Source: Cells. 2021 Apr 1;10(4):784. doi: 10.3390/cells10040784 (PMC8066420; doi:10.3390/cells10040784)
Supplement: Supplementary file 1 [file cells-10-00784-s001.pdf]

Supplementary materials

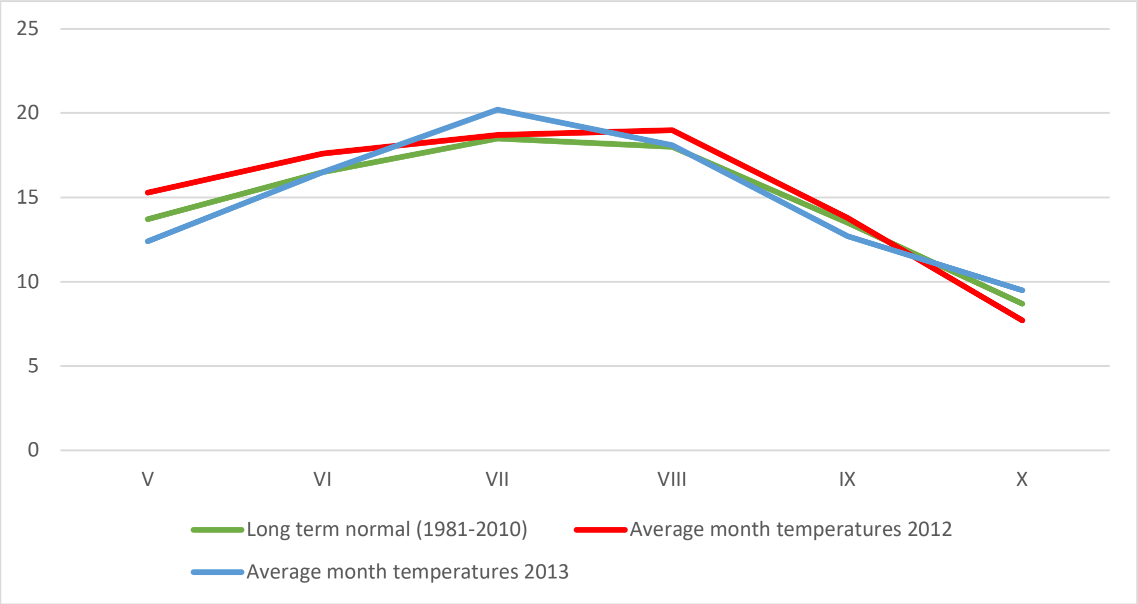

Figure S1. Average temperature profile (°C) at the trial station Troja during the vegetation. period 2012 (**red**) and 2013 (**blue**) compared to the 30-year average (**green**).

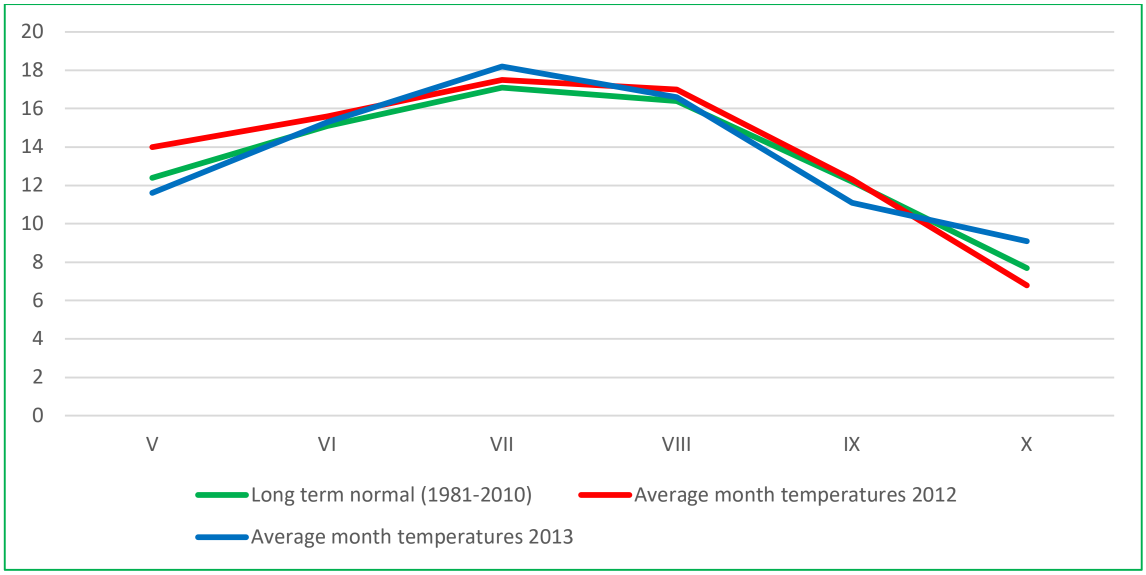

Figure S2. Average temperature profile (°C) at the trial station Svijanský Újezd during the. vegetation period 2012 (**red**) and 2013 (**blue**) compared to the 30-year average (**green**).

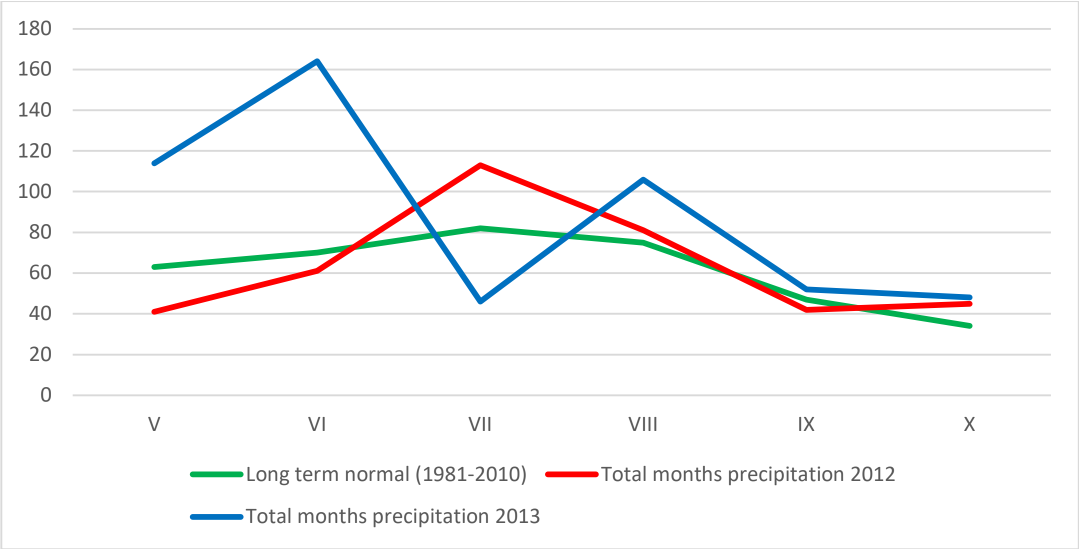

Figure S3. Average precipitation profile (mm) at the trial station Troja during the vegetation period 2012 (**red**) and 2013 (**blue**) compared to the 30-year average (**green**).

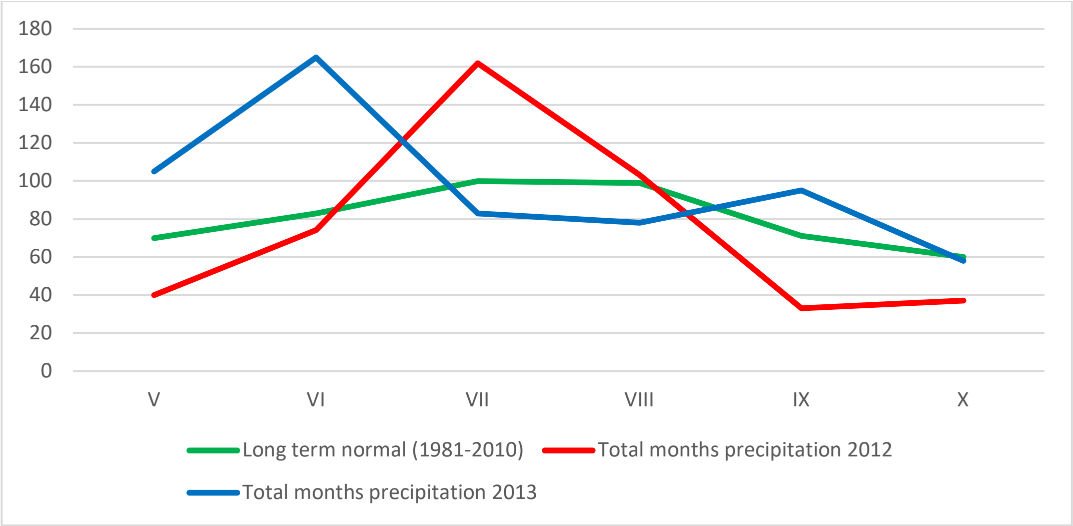

Figure S4. Average precipitation profile (mm) at the trial station Svijanský Újezd during the vegetation period 2012 (**red**) and 2013 (**blue**) compared to the 30-year average (**green**).

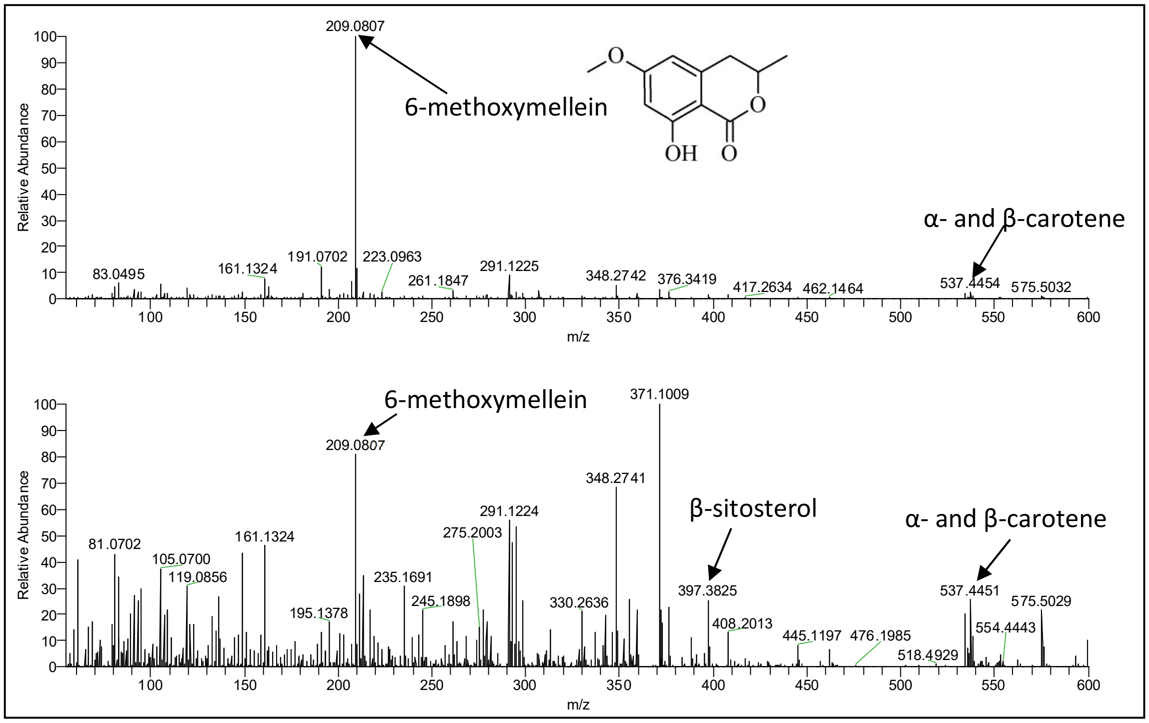

Figure S5. DART-Orbitrap-MS spectra of ethylacetate extracts of Cortina carrot cv. measured in positive ionization mode. Carrot grown in: organic farming system (top), conventional farming system (bottom).

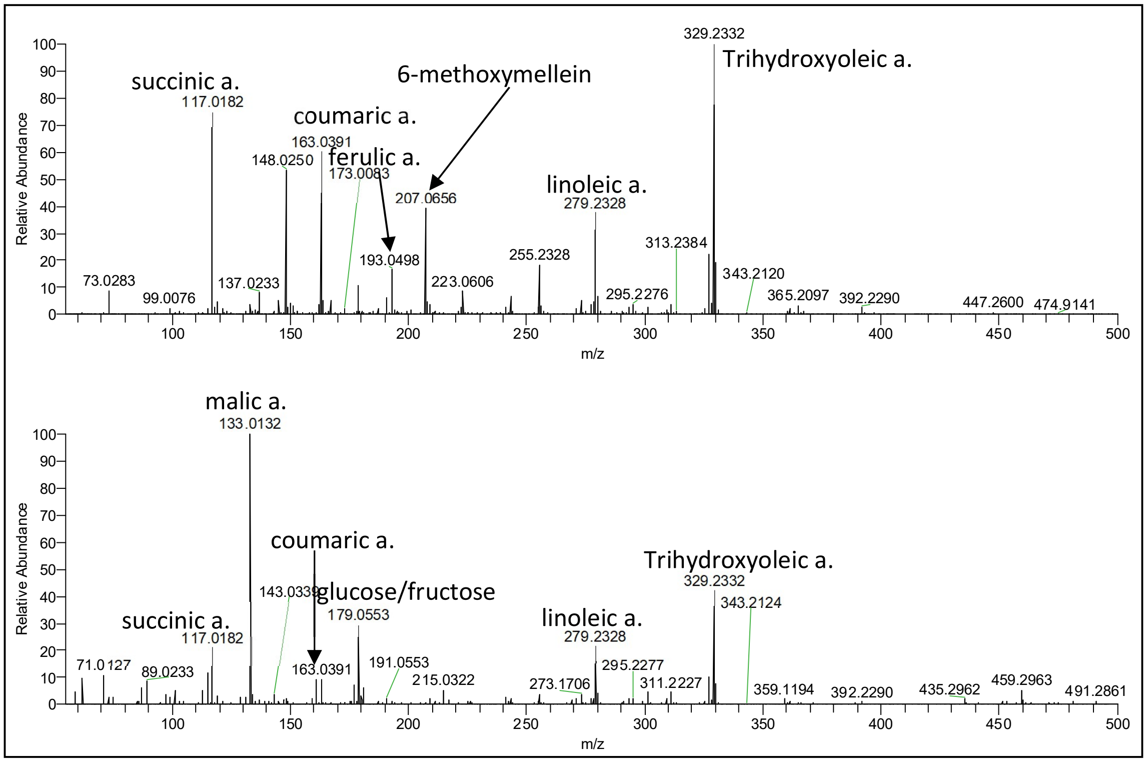

Figure S6. DART-Orbitrap-MS spectra of ethylacetate extracts of Cortina carrot cv. measured in negative ionization mode. Carrot grown in: organic farming system (top), conventional farming system (bottom).

Table 1. Summary of tentatively identified compounds found in carrot extracts.

Identified compound	Molecular formula*	Positive ion mode		Negative ion mode	
		Measured ion	Exact mass	Measured ion	Exact mass
Fumaric acid	$C_4H_4O_4$	-	-	$[M-H]^-$	115.0025
Succinic acid	$C_4H_6O_4$	-	-	$[M-H]^-$	117.0182
Malic acid	$C_4H_6O_5$	-	-	$[M-H]^-$	133.0137
Hydroxybenzoic acid	$C_7H_6O_3$	-	-	$[M-H]^-$	137.0233
Coumaric acid	$C_7H_6O_2$	-	-	$[M-H]^-$	163.0391
Caffeic acid	$C_9H_8O_4$	-	-	$[M-H]^-$	179.0340
Citric acid	$C_6H_8O_7$	-	-	$[M-H]^-$	191.0192
6-Hydroxymellein/ ferulic acid	$C_{10}H_{10}O_4$	$[M+H]^+$	195.0651	$[M-H]^-$	193.0498
Eugenin	$C_{11}H_{10}O_4$	$[M+H]^+$	207.0651	-	-
6-Methoxymellein	$C_{11}H_{12}O_4$	$[M+H]^+$<br>$[M-H_2O+H]^+$	209.0807<br>191.0701	$[M-H]^-$	207.0656
Falcarindiol	$C_{17}H_{24}O_2$	$[M+H]^+$	261.1847	-	-
Linoleic acid	$C_{18}H_{30}O_2$	-	-	$[M-H]^-$	279.2328
Trihydroxy-octadecenoic acid	$C_{18}H_{34}O_5$	$[M+NH_4]^+$	348.2742	$[M-H]^-$	329.2332
α/β-Carotene	$C_{40}H_{56}$	$[M+H]^+$	537.4460	-	-

* Molecular formula was calculated based on the measured exact mass (with the mass error <3 ppm) and isotopic profile.

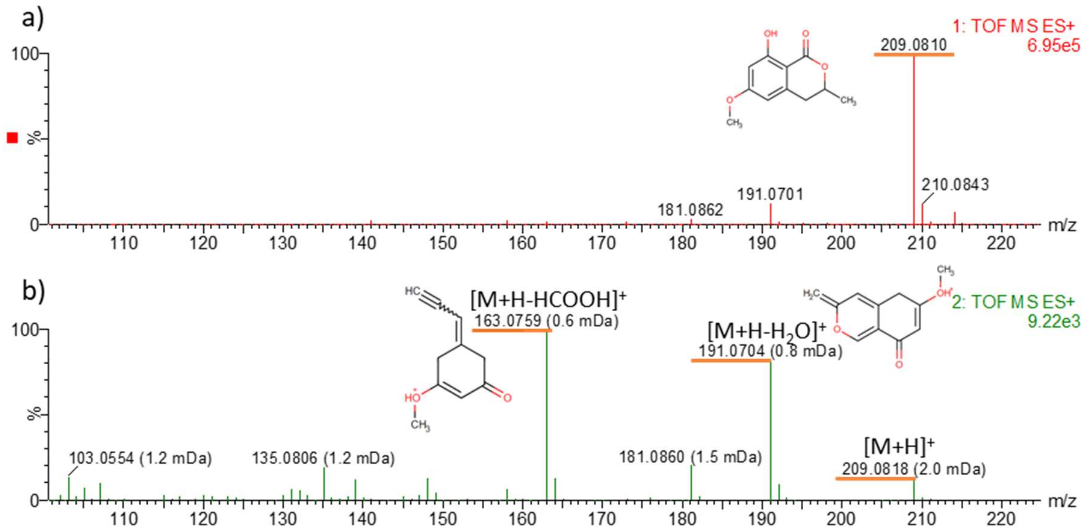

Figure S7. (**a**) LC-HRMS spectrum of 6-methoxymellein (m/z = 209.081) and (**b**) fragmentation spectrum of the parent ion 209.081 (methanolic extract of carrot, positive mode of ionization).
